# Pseudomycetoma of the scalp caused by *Microsporum canis*^☆^^☆☆^

**DOI:** 10.1016/j.abd.2019.07.012

**Published:** 2020-03-19

**Authors:** Ligia Rangel Barboza Ruiz, Clarisse Zaitz, Rute Facchini Lellis, John Verrinder Veasey

**Affiliations:** a Clinic of Dermatology, Faculdade de Ciências Médicas, Santa Casa de Misericórdia de São Paulo, São Paulo, SP, Brazil; b Pathology Laboratory, Hospital da Santa Casa de Misericórdia de São Paulo, São Paulo, SP, Brazil

**Keywords:** Histology, Microsporum, Mycetoma, Tinea, Tinea capitis

## Abstract

Pseudomycetoma is an extremely rare deep mycosis, caused by dermatophytic fungi that penetrate the tissue from infected follicles of tinea capitis. Both clinically and histopathology are similar to eumycetoma, being distinguished through the isolation of the fungus, which in the case of pseudomycetoma can be *Microsporum* spp. or *Trichophyton* spp. genre. We present a 24-year-old man with an exuberant tumor in the occipital region with fistula, whose histopathological examination evidenced grains composed of hyaline hyphae and the culture for fungi isolated the agent *Microsporum canis*. Combined treatment of surgical excision followed by oral griseofulvin for two years was performed, with resolution of the condition.

## Case report

**Figure 1:**
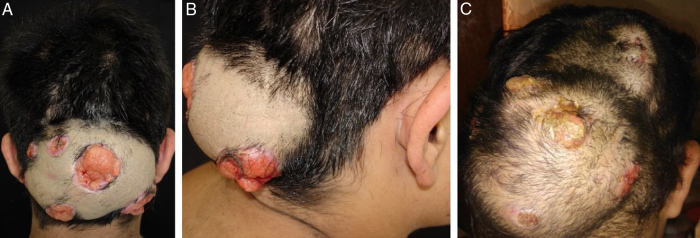
Clinical aspect of pseudomycetoma at occipital lesion. A and B, patient with 14-years-old. C, patient with 24-years-old.

**Figure 2:**
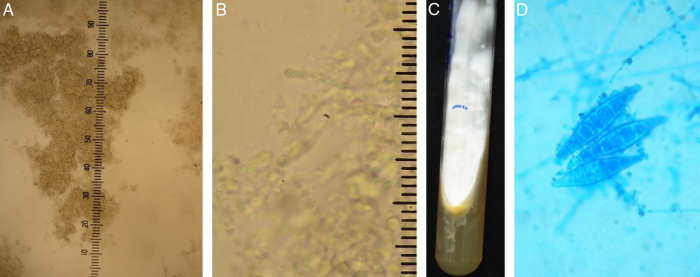
A and B, Direct mycological examination clarified with 20% KOH. ×100 augmentation, showing agglomerates of septated hyphae, and ×400 augmentation, identifying the structures of hyaline septated hyphae. C and D, fungal culture, with white filamentous colony and yellow pigmented agar and microculture with hyaline septated hyphae in the background and three macroconidia in the center (Cotton blue, ×400).

**Figure 3:**
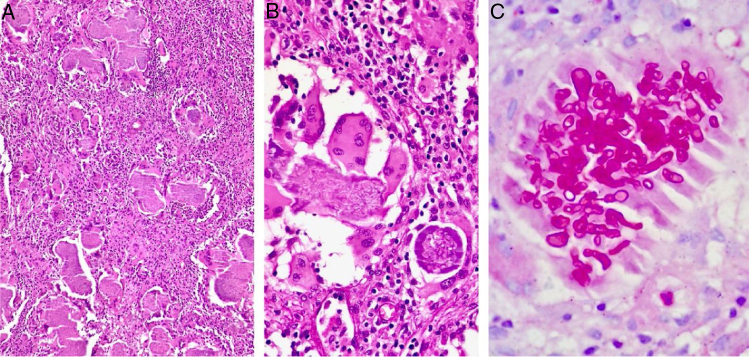
Histopathological examination. A, identifying grains surrounded by inflammatory suppurative process and lymphohistiocytic reaction (Hematoxylin & Eosin, ×100). B, grain composed of septated hyaline hyphae surrounded by eosinophilic material (Splendore-Hoeppli phenomenon) (Hematoxylin & eosin, ×200). C, evidencing the morphology of hyphae (PAS, ×200).

**Figure 4:**
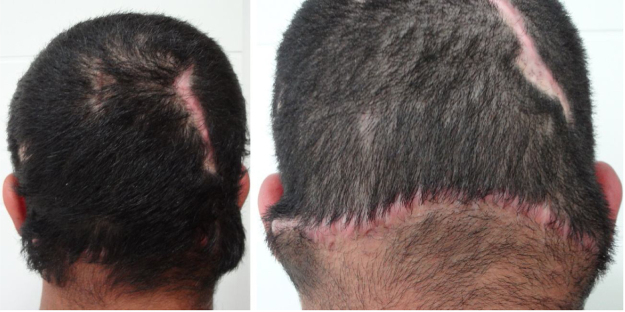
Patient with clinical aspect after treatment.

A 24-year-old immunocompetent male, with tumoral lesion in the occipital area which started at two years of age with areas of alopecia that progressively evolved to a tumor. His first consultation was at age 14, presenting at the clinical examination a hardened tumor with granular fundus ulcerations. He abandoned follow-up, returning ten years later with a considerable increase of the lesion (Fig. 1). Material analysis of ulcer scaling identified at direct microscope examination grains composed of septate hyaline hyphae, and micologial culture isolated *Microsporum canis* (Fig. 2). Histopathological examination revealed at the dermis and hypodermis clusters of septated hyaline hyphae of varied sizes involved by histiocytic Splendore-Hoeppli reaction with numerous multinucleated giant cells of foreign body type, besides neutrophilic exudate, edema and vascular congestion. No fistulated pathways were visualized promoting the continuity between the “grains” and the epidermal surface in the sample (Fig. 3). He denied use of any immunosuppressive medication, presented non-reactive serology for HIV, and had no other comorbidities. Associating the clinical aspect with the complementary tests, the diagnosis of pseudomycetoma by *Microsporum canis* was confirmed. The patient was submitted to surgical excision of the tumor and associated oral griseofulvin, one gram per day for two years. In a one year follow-up after the end of griseofulvin, the patient showed no signs of relapse (Fig. 4).

## Discussion

Chronic inflammatory and invasive forms of dermatophytosis are the result of an intense hypersensitivity reaction to the fungal infection, more frequent in immunocompromised individuals.^1^ The clinical presentations are kerion celsi, Majocchi granuloma and pseudomycetoma.^2^ Pseudomycetoma is an extremely rare mycosis, caused by the penetration of dermatophytes into the tissue from rupture of infected follicular epithelium. Ajello et al.^3^ reported several species of dermatophytes producing grains in tissues, including *Microsporum canis*, *Trichophyton tonsurans* and *T. mentagrophytes*. According to these authors, mycelium aggregates formed by the dermatophytes would be pseudo-granules and the term pseudomycetoma should be applied to this deep dermatophytic infection.^4^, ^5^ The isolated agent in this case was compatible with the most frequent tinea capitis agent in Brazil.^6^, ^7^

Clinical aspects of pseudomycetoma are identical to those of eumicetoma, yet in contrast to mycetomas, pseudomycetomas are more common in the scalp and do not have a history of trauma for its inoculation.^8^ Although the dermatophytic hyphae usually are more delicate than the eumicetoma agents at mycological examinations, the same does not happen in the clusters visualized on the histopathological examination.^7^, ^8^, ^9^ However, there is a difference between the two diseases at histopathology: mycetomas typically have sinus tracts through which fibrinopurulent exudate and grains are readily excreted; by contrast, pseudomycetomas lack sinus tracts.^8^ Therefore, isolation of the agent should be obtained with fungal culture, as here presented. Although more frequent in immunosuppressed patients, there are reports of cases in immunocompetent patients, and the eosinophilic reaction of Splendore-Hoeppli around pseudogranules is present in all cases of pseudomycetoma, highlighting the intense reaction of the organism against the fungus.^4^

The treatment of pseudomycetoma by *M. canis* is surgical excision of the fungal mass, since the systemic antifungal does not reach therapeutic concentrations,^8^ associated with oral griseofulvin^1^, ^6^, ^7^ until clinical and mycological cure.

## Financial support

None declared.

## Authors’ contributions

Ligia Rangel Barboza Ruiz: Approval of the final version of the manuscript; conception and planning of the study; elaboration and writing of the manuscript; obtaining, analysis, and interpretation of the data; effective participation in research orientation; intellectual participation in the propaedeutic and/or therapeutic conduct of the studied cases; critical review of the literature; critical review of the manuscript.

Clarisse Zaitz: Approval of the final version of the manuscript; conception and planning of the study; obtaining, analysis, and interpretation of the data; effective participation in research orientation; intellectual participation in the propaedeutic and/or therapeutic conduct of the studied cases.

Clarisse Zaitz: Intellectual participation in the propaedeutic and/or therapeutic conduct of the studied cases.

John Verrinder Veasey: Approval of the final version of the manuscript; elaboration and writing of the manuscript; critical review of the literature; critical review of the manuscript.

## Conflicts of interest

None declared.
